# Brown Algae *Fucus vesiculosus* in Pasta: Effects on Textural Quality, Cooking Properties, and Sensorial Traits

**DOI:** 10.3390/foods11111561

**Published:** 2022-05-26

**Authors:** Ana Ramalho Ribeiro, Tiago Madeira, Goreti Botelho, Diana Martins, Ricardo M. Ferreira, Artur M. S. Silva, Susana M. Cardoso, Rui Costa

**Affiliations:** 1 Polytechnic Institute of Coimbra, Coimbra Agriculture School, Bencanta, 3045-601 Coimbra, Portugal; madeira_333@hotmail.com (T.M.); goreti@esac.pt (G.B.); ruicosta@esac.pt (R.C.); 2 Research Centre for Natural Resources, Environment and Society (CERNAS), Coimbra Agriculture School, Bencanta, 3045-601 Coimbra, Portugal; 3 Polytechnic Institute of Coimbra, Coimbra Health School, Rua 5 de Outubro—S. Martinho Bispo, Apartado 7006, 3046-854 Coimbra, Portugal; diana.martins@estescoimbra.pt; 4 Coimbra Institute for Clinical and Biomedical Research (iCBR) Area of Environment Genetics and Oncobiology (CIMAGO), Biophysics Institute of Faculty of Medicine, University of Coimbra, 3004-531 Coimbra, Portugal; 5 Center for Innovative Biomedicine and Biotechnology (CIBB), University of Coimbra, 3004-531 Coimbra, Portugal; 6 Clinical Academic Center of Coimbra (CACC), 3004-531 Coimbra, Portugal; 7 LAQV-REQUIMTE, Department of Chemistry, University of Aveiro, 3810-193 Aveiro, Portugal; ric.ferreira@ua.pt (R.M.F.); artur.silva@ua.pt (A.M.S.S.); susanacardoso@ua.pt (S.M.C.)

**Keywords:** *Fucus vesiculosus*, pasta enrichment, sensory analysis, brown seaweed, pasta cooking quality

## Abstract

*Fucus vesiculosus* is a brown seaweed rich in iodine, fucoxanthin, and phlorotannins, all known to be bioactive compounds associated with health-promoting events. The enrichment of a staple food such as pasta with seaweed flour, could convey health benefits without changing eating habits. In this work, *F. vesiculosus* flour (FVF) was incorporated into durum wheat pasta at 1, 5.5, and 10% gradient levels. The pasta enriched with FVF needed additional water during dough formation and required more cooking time, resulting in higher weight gain but also increased cooking loss (observed with 5.5 and 10%). The fracturability of raw pasta decreased for all the FVF pasta, though the cooked firmness and hardness were only affected with the inclusion of 10% FVF. The substitution of wheat semolina with FVF at a 10% level caused an increase in the pasta’s fiber content, which resulted in a more discontinuous protein–matrix structure, as observed at the microscopic level. Untrained consumers were very positive about the overall sensory traits of the pasta with low supplementation levels (1 and 5.5%). About 72% of panelists selected the 1% FVF pasta as their favorite sample. The utilization of FVF in pasta should be targeted at low inclusion levels to cope with the expected texture quality and prevent the impairment of the sensory traits.

## 1. Introduction

A new focus on algae as a food ingredient has been growing in western diets giving consumers awareness of its dietary benefits beyond their macronutrient content [1]. Macroalga or seaweed, is a low-calorie ingredient that is high in protein and low in fat, as well as being a source of hydrocolloids, minerals, vitamins, and bioactive compounds such as antioxidants. The application of macroalga as a health-promoting agent has been studied in several epidemiological trials [2,3].

The potential utilization of seaweed as a functional ingredient in popular foods is a good way to increase its consumption without changing eating habits or cooking methods [4,5]. This food-based approach is also in line with the recommended dietary option requested by nutritionists for achieving an adequate nutrient intake and for preventing and treating disease [6]. The use of seaweed as a dietary ingredient aiming to improve protein or fiber content, or to increase bioactive compounds, has been studied in several foods such as yogurts, quark, bread, and pasta [7,8,9]. Pasta, in all its different shapes, is one of the most important staple foods worldwide, crossing cultural boundaries, and is an accessible and suitable vehicle for health-promoting ingredients. Manufacturing pasta is a relatively well-described process but tailoring pasta to include new, raw ingredients can be a very challenging process [10,11]. The protein–starch network is the backbone of the pasta structure maintaining the texture, cooking performance, and product quality that is valued by consumers [12]. Replacing wheat semolina with other ingredients may disrupt the protein–starch matrix. Also, the source and level of enrichment may cause diverse impacts on the cooked pasta’s properties and organoleptic traits. Different types of dietary fibers (pea flour, guar gum, and inulin) were incorporated into spaghetti pasta up to a 15% level, impacting pasta quality attributes depending on the type (soluble/insoluble) and quantity of the added fiber [13]. Pasta fortification with a 0.5–2.0% microalgae biomass resulted in an increase in the quality parameters and higher acceptance scores by panelists [14]. Also, the utilization of up to 20% of wakame seaweed in pasta improved its nutritional profile and biofunctional properties. The same study stated that pasta with 10% seaweed had better sensory scores compared to pasta with higher seaweed levels, evaluated by regular and native wakame eaters [9].

*Fucus vesiculosus* is a brown macroalga found in the cold temperate waters of rocky intertidal habitats distributed worldwide, with a prevalence in the North Atlantic and the northeast Pacific coastline [15], and is very common in Portuguese seawaters. Besides its protein, mono- and polyunsaturated fatty acids, and vitamin content, this macroalga is considered a rich source of iodine, known for its goiter-preventing effects [16], and fucoxanthin, a xanthophyll brown carotenoid associated with health-preventing effects [17]. Moreover, the potential functional properties of *F. vesiculosus*, identified as having antioxidant, anti-inflammatory, antibacterial, anticancer, antidiabetic, and anti-obesity properties, are mostly conveyed by the dietary fibers and phlorotannins [18,19,20]. The main dietary fibers in *F. vesiculosus* are fucans, alginates, laminarans, and cellulose, with alginic acid being the predominant polysaccharide with levels of up to 59% in a dry basis (d.b.) [21], whereas fucols and fucophlorethols are the predominant phlorotannin compounds found in *Fucus* spp. [22]. Regarding all its nutritional and functional properties, *F. vesiculosus* has recently been suggested as a valuable food ingredient [23] and is commonly available in North Atlantic waters. Paving the way for the expansion of algae in western diets is a growing trend but still very challenging. It requires extensive work in overcoming the technological issues, but most of all in finding successful food–algae combinations that appeal to consumers. Though consumers can be highly motivated to increase algae-enriched products in their diet, flavor acceptance is a determinant for the success of novel foods [24]. Pasta enriched with *F. vesiculosus* can be a very interesting innovative food that combines an accessible and popular meal with a common and nutritionally rich seaweed. The inclusion of *F. vesiculosus* flour was studied in bread dough and resulted in a maximum inclusion of 4% (d.b.) without impairing textural properties [25]. The inclusion of *F. vesiculosus* flour in pasta at high doses has not yet been attempted.

This study aimed to evaluate the effects of replacing durum wheat semolina with *F. vesiculosus* flour in fettuccine. Three increasing concentrations (1, 5.5, and 10%) of *F. vesiculosus* flour were used, targeting a maximum acceptable amount both for technological and organoleptic traits. The impact on the nutritional composition, raw and cooking properties, and microscopic structure were assessed. The sensory properties and willingness to buy Fucus-enriched pasta were also evaluated by an untrained panel.

## 2. Materials and Methods

### 2.1. Materials

Durum wheat semolina (DWS) was purchased from Cerealis (Maia, Portugal) milling plus 425 µm. Dried and milled *F. vesiculosus* flour (FVF) (<0.25 mm) was supplied by ALGAplus (Ílhavo, Portugal).

### 2.2. Pasta Production

Four experimental pasta formulations were prepared. A control pasta (CTRL) similar to commercial pasta containing only DWS and Fucus-fortified pasta formulated by replacing DWS with 1 (F1), 5.5 (F5), and 10 (F10) g of *F. vesiculosus* flour per 100 g of flour-based mixture. Semolina and FVF were mixed until a homogeneous mixture was obtained. After homogenization of DWS and FVF, each mixture was mixed with gradient levels of of 40 (CTRL), 40 (F1), 48 (F5), and 56 (F10) mL of water per 100 g mixture, as determined for each formulation by preliminary trials. The dough was extruded as lasagna sheets with a Pasta maker machine (Philips Avance Collection HR2354/12, Amsterdam, the Netherlands) and cut as fettuccine with a manual pasta machine (ITALIA, Casa International NV, Olen, Belgium). The experimental pasta was dried in a ventilated oven (Falc Oven Model STE-F 52, Treviglio, Italy) at 55 °C until it reached a moisture level of approximately 11%. Pasta samples were packed in plastic film and stored at room temperature until further analysis.

### 2.3. Physicochemical Analysis

Moisture content was determined by oven-drying at 103 °C using constant weight Method 44-15.02 (Moisture—Air-Oven) [26]. Ash was measured by incineration in a muffle furnace at 550 °C for 6 h and gravimetric quantification according to Method 08-01.01 [26]. Total protein content was estimated by determination of elemental nitrogen content by thermal conductivity using a TruSpec 630-200-200 CNHS analyzer (LECO, St. Joseph, MI, USA), multiplied by a conversion factor of 5.83, which is specific for products based mainly on wheat [27]. Total dietary fiber was determined by an enzymatic gravimetric method, AAAC Method 991.43 [28], using megazyme total dietary fiber assay kit (Bray, UK). Determination of approximate water hydration capacity of durum wheat semolina and *F. vesiculosus* flour was made according to AACC Method 56-30.01 (Water Hydration Capacity of Protein Materials) [26].

### 2.4. Pasta Cooking Quality

The optimum cooking time was determined according to AACC-approved Method 66-50.01 (Pasta and Noodle Cooking Quality—Firmness) [24]. Briefly, 20 g of pasta was cooked in 300 mL of demineralized boiling water. At 30 s intervals, pasta was removed and pressed between two glass plates. Optimal cooking time (OCT) was determined when the central core disappeared when squeezed between two glass plates. The cooked pasta was transferred into a beaker with water at room temperature before testing. All tests were performed in triplicate.

Weight gain was determined as the weight increase of pasta over dry pasta weight (Equation (1)):

(1) $$\mathrm{Weight}\;\mathrm{gain}\;(g/g)=\frac{\mathrm{weight}\;\mathrm{of}\;\mathrm{cooked}\;\mathrm{pasta}\;\left(g\right)\;-\;\mathrm{weight}\;\mathrm{of}\;\mathrm{dry}\;\mathrm{pasta}\;\left(g\right)}{\mathrm{weight}\;\mathrm{of}\;\mathrm{dry}\;\mathrm{pasta}\;\left(g\right)}$$

Cooking loss was evaluated by determining the number of solids lost in cooking water [29]. After cooked, each sample was rinsed with 50 mL of filtered water in a Buchner funnel and drained for two min before weighing. Cooking and rinsing water were recovered. The material leached into the cooking water was estimated by evaporating the cooking and rinsing water to dryness in an air oven at 105 °C. The residue was weighed and reported as a percentage of the original pasta sample. All tests were performed in triplicate.

Cooking loss was determined as (Equation (2)):

(2) $$\mathrm{Cooking}\;\mathrm{loss}\;\left(g/g\right)=\frac{\mathrm{weight}\;\mathrm{of}\;\mathrm{cooking}\;\mathrm{water}\;\mathrm{dried}\;\mathrm{residue}\;}{\mathrm{weight}\;\mathrm{of}\;\mathrm{dry}\;\mathrm{pasta}}$$

### 2.5. Texture Analysis

The texture of raw and cooked pasta was determined using a TAXT2i Texture Analyzer equipped with a Windows version of Texture Expert software package (Stable Micro Systems, Surrey, UK). Raw pasta fracturability was measured using a three-point bend test. A single pasta strand with five-centimeter length was placed perpendicular to two triangular perplex supports separated by a distance of 4 cm and cut with a light knife-blade attachment (thickness 1 mm). Each test was replicated with new pasta strands at least five times. The maximum force value (g) at break was recorded as raw pasta fracturability.

Testing of cooked pasta was performed immediately after cooking to minimize changes resulting from storage in a liquid medium. Cooked firmness was determined according to AACC standard 66-50.01 [26]. Four fettuccine-shaped pasta strands were placed in the center of the measuring area and cut with a light knife-blade attachment (thickness 1 mm). The maximum force value was recorded as pasta firmness. Test parameters were 2 mm/s pre-test speed, 3.0 mm/s test speed, and 10 mm/s post-test speed; the distance was adjusted to a maximum of 1 mm and 15 g was fixed as trigger force.

The cooked pasta hardness was determined using a 35 mm diameter flat-ended cylindrical probe (P/35, Stable Micro Systems). Two fettuccine strands (2 cm) were 70% compressed at a speed of 1.0 mm/s. The force at 70% compression was taken as a measure of the firmness (N) of the fettuccine strands. The probe was moved to its original position at the same speed. The height and width of strands were measured for each type of pasta and values were adjusted in the Texture Expert program.

### 2.6. Pasta Color

The color pasta parameters were measured in an uncooked lasagna sheet, with a colorimeter (model CR200b, Minolta Corp., Ramsey, NJ, USA), and the L*, a*, and b* coordinates from CIELab system were recorded. Color was determined at least in triplicate measures. To estimate perceptible color differences (∆E*) among pasta treatments, the CIE76 formula (based on the Euclidian distances between colors in CIELab space) was calculated by Equation (3).

(3) ΔEab*=[(L2*−L1*)2+(a2*−a1*)2+(b2*−b1*)2] 12

where ∆E* is the total color difference between the sample and the control, L_2_*and L_1_*are the lightness of the sample and control, a_2_* and a_1_* are the redness of the sample and control, and b_2_* and b_1_*are the yellowness of the sample and control, respectively. Following the definition of the CIELab color space and Mahy [30], distances between colors were considered as being indicative of either an “irrelevant perceptual difference” (ΔE* < 1), a “slight perceptual difference” (1 < ΔE* < 2.3), or a “clear perceptual difference” (ΔE* > 2.3).

### 2.7. Microscopic Structure of Cooked Pasta

The microstructure of CTRL, F1, F5, and F10 pasta cooked to OCT was observed using bright-field light microscopy. Pasta sections (8 µm) were stained for 10 min with fast green (Sigma Aldrich Co., St. Louis, MO, USA) and for 1 min with lugol (Fluka, Buchs, Switzerland) [31,32]. Bright-field images were acquired using the Nikon Eclipse Ci-L (Nikon, Minato, Japan). Observations were made with plan achromatic objectives of 10 and 20 with a fixed optical zoom of 10, resulting in a total magnification of 100 and 200.

### 2.8. Sensory Evaluation of Pasta Products

Sensory evaluation was carried out in an acclimatized room equipped with individual booths. Ratings for preference test were conducted [33] using a sensory panel composed of 71 untrained volunteers, mainly undergraduate students and professionals working at Coimbra Agriculture School. Written informed consent was obtained from all volunteers. Most respondents were female (71%). Age range was between 18 and 60; 16% were less than 20 years, 67% were between 20 and 30 years, and 17% were over 30 years old. The panel was characterized by high consumption of pasta products (90% eat pasta at least once a week) and low consumption of seaweed (62% eat twice a year maximum and 29% never consume). Most panelists (88%) rated as adequate or very adequate the food supplemented with seaweed and 91% considered themselves motivated or very motivated to try new food products.

Each experimental pasta was cooked to its optimal cooking time and immediately placed in white cups and covered. Cooked pasta samples were presented to the panelists sequentially in three-digit-coded white cups under normal white lighting. Each panelist received the control pasta and the sequence of three samples (experimental pasta with 1, 5.5, and 10% of *F. vesiculosus*) in a balanced random order and rated, for all samples, the intensity of sensory attributes on a 9-point scale ranging from extremely dislike (1) to extremely like (9 points). The selected attributes were the visual aspect, odor, taste, texture, and overall acceptance. The panelists also indicated their favorite and their least-favorite samples and expressed their purchase intention on a 5-point scale from 1—certainly would not buy—to 5—certainly would buy.

### 2.9. Statistical Analysis

All measurements were done at least in triplicate and data relative to instrumental determination of cooking quality were subjected to an analysis of variance using the IBM SPSS Statistics (v25, IBM, Armonk, NY, USA). ANOVA was followed by Tukey’s post hoc test. Non-parametric Kruskal–Wallis test was used when experimental data did not meet ANOVA assumptions. Significance level used was *p* < 0.05.

## 3. Results and Discussion

This study assessed the impact of incorporating the macroalga *F. vesiculosus* in a pasta product on its different properties: texture, cooking performance, microstructure properties, and consumer sensory acceptance. Three different concentrations of FVF (1, 5.5, and 10%) were used to test an optimal combination between the consumer acceptance level and the possible technological constraints.

Before presenting the results of the dried and cooked pasta, it is worth mentioning that during pasta preparation, a higher water addition to the pasta dough was needed with increasing levels of FVF incorporation. An increased water absorption of doughs with seaweed powder, as previously described, is possibly due to the higher water affinity of the seaweed’s fibers, measured as the water retention capacity [34,35]. Moreover, due to seaweed’s smaller particle sizes, the available surface area for hydration is enlarged. In the present work, the measured approximate water hydration capacity was 5.21 mL∙g^−1^ and 1.74 mL∙g^−1^ for FVF and semolina, respectively, which may explain the need for the additional water in the pasta dough.

### 3.1. Proximate Composition

The proximate composition of the raw experimental pasta is described in Table 1. The moisture values after the drying process were within the recommended values for pasta [36], with the water activity below 0.5 for all the samples (data not shown). The FVF used in the experimental pasta contained 12% moisture and approximately 11.7% (dry basis) protein, which is similar to the values reported by Lorenzo et al. [37] for *F. vesiculosus* captured on the Atlantic coast (13%, d.b.). The semolina used as the control contained the same protein concentration (12%), therefore the replacement of semolina with FVF did not modify the total protein levels of pasta (Table 1). The protein content was estimated using a nitrogen-to-protein conversion factor of 5.83, corresponding to a whole-wheat kernel [27]. However, the most adequate factor would lie between this one and the suggested values for seaweed, ranging from 4.3 *for F. vesiculosus* on a Norwegian sample [38] to 5 for other seaweed [39]. The ash content of FVF was 25.7% d.b., which is very high compared with the approximately 1% content of durum wheat semolina. So, as expected, ash values were significantly higher (*p* < 0.05) with increasing levels of seaweed incorporation in pasta. The same was expected for the fiber content since the FVF fiber content was 41.4%; however, this was only observed at the maximum seaweed concentration (F10) when compared to the other formulations. This could be explained by the high variability of the results obtained with the method used for fiber analysis, which presented a large standard deviation, preventing the observation of changes that are smaller than the experimental error of the analysis.

### 3.2. Pasta Quality and Texture

The results of the texture analysis and cooking properties of the experimental pasta are reported in Table 2. The inclusion of FVF modified the textural properties and cooking behavior of pasta. The firmness of raw pasta decreased as semolina was replaced by FVF, observed even with only a 1% inclusion of seaweed. However, no significant differences were registered between 1% and 5.5% FVF. In contrast, Fradique et al. [14] described an increase in raw pasta firmness with a 2% microalgae *Chlorella vulgaris* and *Spirulina maxima* enrichment, a fact that the authors attributed to the higher protein content (freeze-dried *C. vulgaris*—38% protein db; freeze-dried *S. maxima*—38% protein d.b.) and lower water uptake during dough formation, which also diverged from our results. In the present work, a lower gluten and higher fiber content can explain the raw firmness decrease, even if it was only detectable and significant in F10 pasta.

Cooking performance and pasta texture are very important traits concerning pasta quality and consumer acceptance. In cooked pasta, the firmness and hardness presented no differences between the control, 1%, and 5.5% FVF pasta samples. Yet, these parameters decreased with the addition of 10% macroalga, resulting in a “softer” pasta. The optimal pasta structure and cooking quality are dependent on the formation of gluten and the gluten–starch matrix, varying with the protein and starch sources [40,41]. The protein content among the experimental pastas was similar; however, in the FVF pastas, the formation of the gluten network was probably affected by the included protein profile and the higher fiber levels, which altered the cooked pasta’s firmness and expected quality. A weakening of the gluten network in pasta with the brown seaweed *Sargassum marginatum* at levels beyond 2.5% was suggested as an explanation for the decrease in the shear force [42]. Although observed in a different matrix, in wheat bread *F. vesiculosus* powder also precluded the formation of a gluten network, may be perhaps owing to the competition for available water or because of steric hindrance [25]. Laleg et al. [31] reported similar results but used a legume, faba protein, in pasta and hypothesized that the increase in the proportion of faba protein acted as a dilution agent of gluten mainly at low drying temperatures (55 °C). The increase in the fiber content causes a disruption in the protein–starch network [43] and consequently a reduction in pasta firmness. In contrast, an increase in the protein concentration was reported to also increase firmness (reviewed by [44]). In the present work, the total protein did not increase, but the gluten proteins were proportionally fewer, causing the referred dilution effect and a weakening of the pasta structure.

During pasta cooking, a mass transfer occurs with the water uptake by the pasta dough and the dispersion of solids into the cooking medium. Weight gain due to water absorption and cooking loss at the optimum cooking time, are considered indices of pasta quality [45,46,47]. The main processes during pasta processing are the formation of gluten and starch gelatinization, both of which are water-dependent processes [10]. The incorporation of seaweed flour increases the fiber content in the pasta dough; fiber competes for water uptake with the protein–starch matrix [48]. Consequently, the inclusion of seaweed resulted in an increase in the weight gain of the cooked pasta samples, independently of the FVF concentration used (Table 2). In the pasta enriched with wakame of up to 30%, the cooked weight increased with increasing seaweed levels [9]. Gallo et al. [49] studied the effects of fiber in the microstructure of commercial pasta by comparing durum wheat semolina and whole durum wheat semolina spaghetti samples. They found that the presence of fiber affected the water mobility at the molecular level due to the interactions of the water protons with fiber, starch, and the protein matrix, resulting not only in a higher water absorption during cooking but also in a structure loss. Mercier et al. [44], in a meta-analysis comparing enriched pasta, reported that high fiber enrichment (>15%) led to a reduced weight increase during cooking. However, the same study states that a low fiber enrichment (<15%) in pasta dried at low temperatures (≤60 °C), as in the present work, caused higher weight increases during cooking. In these conditions, fiber may weaken the pasta structure and absorb more water.

The cooking loss proportionally increased with the higher FVF content in pasta, a fact that may be a result of the decreasing gluten content in spaghetti, which facilitates the loss of soluble and non-soluble solids into the cooking water [40]. Note that the structure and firmness of pasta are dependent on the degree of protein coagulation, starch hydration, and gelatinization during the cooking process [10]. Thus, macroalgae fiber aggregates water (increasing water uptake) but simultaneously disrupts the pasta structure, resulting in a net increase in the loss of solids during cooking. The utilization of legume protein (faba and pea) in pasta with its increased fiber content, was shown to induce the formation of cracks and discontinuities in pasta strands [32,50] with a concomitant increase in a loss of solids [31]. Nevertheless, the cooking loss values obtained in this work were all below 8%, which is still considered an acceptable value for good quality pasta [51].

The optimal cooking time was 9 min for the CTRL, 13 min for the F1, 14 min for the F5, and 15 min for the F10 samples, increasing with a higher content of FVF, determined as a delay in the disappearance of the inner core of the macroalga-enriched pasta. However, it was observed that the pasta’s outer surface was overcooked but the inner center was still undercooked, resulting in an overall softer texture. Seaweed fiber may also form a barrier to water uptake delaying starch hydration in the inner core. The extra cooking time can cause an increase in the cooking loss. In contrast, several works showed a reduction in the OCT with a variety of pasta enrichments [32,44]; however, only Sęczyk et al. [52] described a longer OCT of pasta enriched with carob flour of up to 5%.

### 3.3. Microscopic Structure

**Figure 1:**
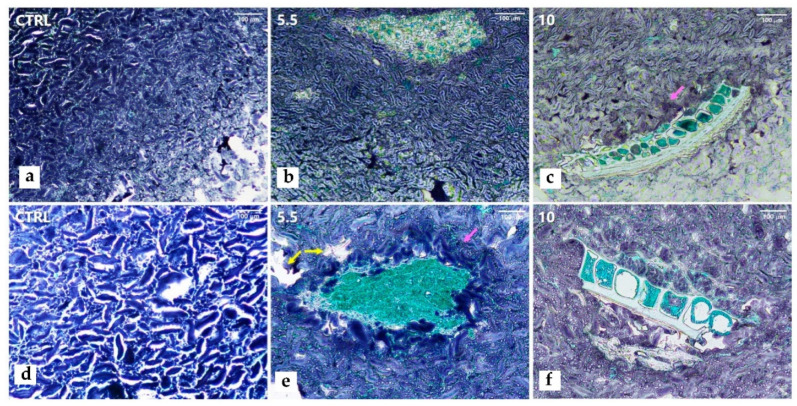
Light microscopy of cooked semolina pasta (**a**,**d**—CTRL) and pasta enriched with 5.5% (**b**,**e**) and 10% (**c**,**f**) of *F. vesiculosus* flour, using 100× (top row) and 200× (bottom row) magnification.

The microscopic structures of the FVF 5.5 (Figure 1b,e), 10 (Figure 1c,f), and CTRL (Figure 1a,d) cooked pasta are presented in Figure 1 using two magnifications (a–c—100×, d–f—200×). The images represent the different sections of the pasta, that is, the core, intermediate, and surface regions (Figure 1—100: a–c). It was possible to observe structural changes from the external region to the core in all samples. The inner center (Figure 1a top left) presented a more compact structure compared to the peripheral area, corresponding to a decreasing moisture gradient, as reported in other works [31,32]. The starch granules (dark blue) were more defined and elongated in the center compared with the border, which had a more diffused and unorganized structure, with the starch granules losing their shape and being less stained. Larger white spaces around the starch granules and darker-blue stains in the core pasta starch could be observed in the CTRL (Figure 1d), which may indicate limited granule swelling and gelatinization due to the lower hydration levels [53] in the pasta dough and less cooking time. In contrast, in the pasta with 5.5 and 10% FVF, the core starch granules were less dark and the protein network (light blue) more visible (Figure 1e,f), possibly induced by the higher starch gelatinization due to longer cooking times, whereas the CTRL had a more preserved structure [53]. In the semolina pasta (CTRL), a more continuous and denser network was observed compared to a more discontinuous matrix of the FVF pasta with larger pores (Figure 1e—yellow arrows). Macroalga fibers were visible in the formulated pasta samples, increasing in proportion to the higher FVF content. The fibers were randomly distributed between the core, intermediate, and border regions. It was previously hypothesized that the introduction of fiber particles weakens the pasta structure, disrupting the protein–starch network [13] due to a dilution of the gluten proteins that interferes with the gluten development [54], allowing high water absorption. In contrast, lower core hydration was also reported [55] since fiber may create a barrier to moisture, limiting the starch swelling and increasing the optimal cooking time. This was observed in the FVF pasta, where darker starch granules were present next to FVF fibers (Figure 1c,e—pink arrows). The differences reported seem to be dependent on the type of fiber used [44]. The macroalga fiber probably delayed the water migration and raised the OCT with the increasing levels of FVF in the pasta. This longer exposure to hot water affected the microscopic structure of the FVF pasta resulting in a less compact structure, and consequently an increased loss of solids.

### 3.4. Color

Consumer acceptance of a food product is highly conditioned by its appearance. The color and visual image are the first attributes evaluated by consumers during purchasing and, as has been well described, may influence the flavor perception and food acceptability [56,57]. Semolina pasta exhibits a typically yellow color resulting from the lutein and beta-carotene pigments. In brown seaweed, such as *F. vesiculosus*, fucoxanthin, a xanthophyl carotenoid, exists abundantly giving algae its brown or olive-green characteristic color. The addition of FVF flour to pasta expectedly modified its pigmentation from yellowish to brown. A clearly perceptible difference between the control and the seaweed pasta color was confirmed according to the ΔE* values (ΔE* varied between 12.4 (ΔE* CTR—F1), 118.9 (ΔE* CTR—F5), and 136.5 (ΔE* CTR—F10). The F1 pasta presented a slightly darker pale color, whereas the F5 and F10 pasta developed a dark-brown pigmentation. The resulting stronger brown color was not homogeneous; several dark spots were visible. The main differences between the control samples were clearly observed in the lightness parameter, which was significantly lower in the *F. vesiculosus* pasta in comparison with the CTRL pasta (Table 3). Enrichments generally decrease pasta brightness [44], which can negatively impact consumer acceptance. The darker color of most enrichment ingredients, non-enzymatic browning, and carotenoids oxidation were stated as being the main factors causing the lower brightness. The experimental pasta with 5.5 and 10% FVF presented a combination of more intense green and yellow colors (negative a* and positive b* values, respectively), significantly different from the CTRL and F1 pasta, both with a paler pigmentation. Although the F10 had almost twice the seaweed flour concentration compared with the F5, this difference was not evident in the measured color parameters. The CTRL’s color measurements were within the range of values described by Mercier et al. [44], except for the a* parameter (−2.3 to 9.6), which was close to the minor value.

### 3.5. Sensory Analysis

**Figure 2:**
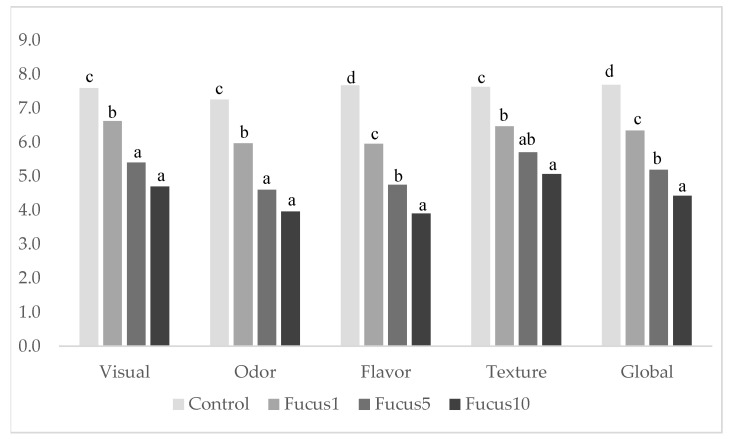
Sensory scores of semolina pasta (CTRL) and pasta enriched with F. vesiculosus (F1, F5, F10) by an untrained panel. Attribute intensity was rated on a 9-point scale ranging from extremely dislike (1) to extremely like (9 points). Different superscript letters correspond to significant differences within each column.

Pasta’s sensory characteristics are related to its textural properties and are mostly influenced by changes occurring during cooking. The most significant correlations found regarding pasta’s overall quality were associated with its appearance, flavor, and texture, properties highly valued by consumers and essential to its quality [44]. The results of the sensory evaluation are reported in Figure 2. Panelists preferred the CTRL sample and the pasta with 1% FVF, both with positive scores above 7 (moderately liked) and above 6 (slightly liked), respectively. Other studies showed that low inclusion levels (2–3%) of blue-green microalgae in pasta even improved the overall acceptability [14,58]. In turn, higher inclusions of FVF (5.5 and 10%) were the least accepted pasta and were similarly rated with low scores regarding the odor, flavor, and texture criteria. Overall, flavor and odor were the most affected attributes with macroalgae inclusion in the pasta. A sea smell, considered pleasant at low concentrations, was frequently referred to in the FVF pasta. Peinado et al. [59] related the volatile compounds, which usually characterize the typical aroma and flavor, of *F. vesiculosus* aqueous extracts to herbal and fishy notes.

Concerning pasta texture, a sandy/granular feel was often referred to by panelists as being both present and also unpleasant in the pasta with 10% FVF. Excessive cooking times, necessary for the pasta’s core appearance, caused an outer softer texture that also contributed to the general dislike of the pasta with higher FVF content. Similar findings have been reported in the literature either with high levels of pasta enrichment or with the use of seaweed as a food ingredient. Mercier et al. [44] described a general negative correlation between the enrichment level with several ingredients (protein concentrates and isolates, legumes, oilseed, and microalgae and fruit extracts) and pasta’s overall quality, appearance, and flavor, which was reflected in its lower acceptance. The reluctance to accept high seaweed contents was also observed in native seaweed eaters. Prabhasankar et al. [9] observed that native eaters of wakame revealed a decreasing appreciation of this seaweed with contents ranging between 5 and 30%, with 10% being the acceptance limit.

The alteration of sensorial traits in several food products using *F. vesiculosus* as an ingredient has also been highlighted by other authors, e.g., the addition of *F. vesiculosus* ethanol extracts to milk and yogurt at 0.25% and 0.5% respectively, impaired typical sensory traits and overall acceptability [60,61]. Negative sensory properties were associated with a fishy taste and an off flavor. In turn, the flavor of fish mince cakes with added antioxidant dietary fiber from *F. vesiculosus* was found to be no different from the control samples with a 1% but not those with a 2% fiber inclusion [62]. Fish-based foods may be more adequate for FVF inclusion than other foods due to their natural fishy taste. The combination of food carrier and beneficial ingredients is extremely important since consumers fear the off flavor caused by a functional ingredient when there is no natural link to the carrier product [63].

Between the three FVF enriched pasta samples, the F1 was selected as the preferred sample by 72% of panelists, whereas 19% preferred the F5 and 9% preferred the F10. Accordingly, buying intention decreased with increasing concentrations of FVF in the pasta (F10—2; F5—2.7; F1—3.6; CTRL—4.5). The results indicated that consumers were willing to consume macroalga-enriched pasta but with low incorporation levels of FVF. Concerning buying preferences, 55% of participants would hypothetically buy the F1 pasta and 35% the F5 pasta, suggesting that a potential niche market exists for seaweed-enriched pasta. Enrichment clearly changed the pasta’s characteristics and sensory traits and consumers are not willing to compromise taste over the potential health benefits [64].

## 4. Conclusions

This exploratory study showed that the substitution of durum wheat semolina pasta with *F. vesiculosus* flour affected the cooking quality parameters, particularly at a 10% level. These effects were mainly caused by the dilution effect of gluten proteins, with the increased seaweed fiber content disrupting the gluten–starch matrix, an event also reflected in the modified microstructure integrity. Fiber takes on water limiting its absorption by the surrounding starch, which delays cooking, leading to long cooking times and, consequently, high weight gain and high solids loss. Raising the FVF to 10% resulted in weaker quality indicators, such as more brittle uncooked and cooked pasta, and impaired sensory traits.

Targeting pasta fortification with low seaweed levels will be more suitable for consumers, such as the Portuguese, who are not used to eating macroalgae in their dietary pattern. For all measured attributes, consumers’ preferences decreased with increasing seaweed levels in the pasta samples, though the panelists positively rated the organoleptic properties of the 1 and 5% experimental pasta, but not the 10% FVF pasta. Considering that FVF is a very rich source of several minerals, including iodine, pasta with 1% FVF could contain between 130 and 730 µg of iodine [65] per 100 g of raw pasta, and for the 5% Fucus pasta, between 650 and 3650 µg of iodine per 100 g of raw pasta. These are very high iodine intake levels according to the recommended daily intake of 150 µg a day [66]. The consumption of *F. vesiculosus*-enriched pasta would clearly contribute to the intake of daily or weekly iodine requirements.

In future works, *F. vesiculosus* flour may be tested in other foods, namely in seafood-based products due to its fishy odor. Also, it would be interesting to extend the consumer panel to older age ranges and consumers living in other countries in order to include the diversity of eating patterns, which could enable wider prospects for the acceptability of FVF-enriched pasta.

## Figures and Tables

**Table 1:** Proximate composition of raw semolina pasta (CTRL) and pasta enriched with *F. vesiculosus* (F1, F5, F10) (g∙100 g^−1^ pasta d.b.).

Pasta	Moisture	Protein	Total Fiber	Ash
CTRL	11.59 ± 0.01 ^a^	11.75 ± 0.01 ^a^	4.12 ± 0.48 ^a^	0.75 ± 0.03 ^a^
F1	12.17 ± 0.10 ^a^	11.78 ± 0.12 ^a^	3.86 ± 0.06 ^a^	1.04 ± 0.10 ^b^
F5	12.31 ± 0.36 ^a^	12.21 ± 0.14 ^a^	4.52 ± 0.34 ^a^	1.84 ± 0.04 ^c^
F10	11.83 ± 0.09 ^a^	11.82 ± 0.59 ^a^	7.00 ± 0.06 ^b^	2.89 ± 0.07 ^d^

Values are average of at least three determinations. Different superscript letter corresponds to significant differences within each column (*p* < 0.05).

**Table 2:** Texture and cooking properties of semolina pasta (CTRL) and pasta enriched with *F. vesiculosus* (F1, F5, F10).

Pasta	Firmness Raw (g)	Firmness Cooked (g)	Hardness (g)	Weight Gain (g/g)	Cooking Loss (g/g)
CTR	198 ± 31 ^c^	428 ± 66 ^b^	5179 ± 479 ^b^	1.45 ± 0.11 ^a^	3.78 ± 0.42 ^a^
F1	123 ± 33 ^b^	396 ± 26 ^ab^	5479 ± 517 ^b^	1.75 ± 0.06 ^b^	4.55 ± 0.20 ^a^
F5	117 ± 15 ^b^	422 ± 53 ^b^	5293 ± 484 ^b^	1.88 ± 0.10 ^b^	6.09 ± 0.23 ^b^
F10	77 ± 18 ^a^	360 ± 30 ^a^	4537 ± 311 ^a^	1.88 ± 0.06 ^b^	7.54 ± 0.35 ^c^

Values are average of at least three determinations. Different superscript letters correspond to significant differences within each column (*p* < 0.05). OCT (min): CTRL—9; F1—13; F5—14; F10—15.

**Table 3:** Color parameters (L*, a*, b*) of uncooked semolina pasta (CTRL) and pasta enriched with *F. vesiculosus* (F1, F5, F10).

Sample	CTRL	F1	F5.5	F10
L* lightness	78.7 ± 2.1 ^c^	66.4 ± 1.8 ^b^	40.8 ± 2.7 ^a^	38.2 ± 3.4 ^a^
a*	−3.1 ± 0.4 ^a^	−2.7 ± 0.7 ^a^	−112.8 ± 5.8 ^b^	−131.2 ± 30.7 ^b^
b*	27.8 ± 3.7 ^a^	27.3 ± 1.1 ^a^	53.7 ± 11.4 ^b^	51.8 ± 13.3 ^b^

Values are average of at least three determinations. Different superscript letters correspond to significant differences within each line (*p* < 0.05).

## Data Availability

The data presented in this study are available in article.
